# Observations on the Position of a Patient after the Operation for Hernia

**Published:** 1821-06

**Authors:** R. Wade

**Affiliations:** Member of the Royal College of Surgeons, and Apothecary to the Westminster General Dispensary.


*Clifton; April* 8, 1821.

---

*Observations on the Position of a Patient after the Operation for Hernia.* By R. Wade, Member of the Royal College of Surgeons, and Apothecary to the Westminster General Dispensary.

HOWEVER unimportant the following communication with regard to position after the operation for strangulated hernia, may at first sight appear, yet its highly beneficial effects, in the case I am about briefly to relate, were so evident, and the subject but so very slightly noticed by writers on Hernia, that I am induced to request a page in your Journal for its insertion, with a view to recommend a more general adoption of the treatment.

The patient, Mrs. ——, aged 58, and residing at No. 4, Crown-court, St. James's, had a strangulated femoral hernia, attended by the usual symptoms; and it will be sufficient for my purpose to state, that the remedies usually had recourse to in such cases were employed in the first instance, without the slightest diminution of urgent symptoms; when, on the fourth day of strangulation, the operation was performed by Mr. A. Copland Hutchison, surgeon to the Dispensary, with as little injury to the protruded parts as their adhesions to the sac would permit. Soon after the operation, the patient's bowels were freely relieved. Her vomiting ceased, and no bad symptoms occurred, until the third day after the operation, when the wound became painful,—the parietes of the abdomen again very tender to the touch,—occasional vomiting,—a quick, small, and intermitting pulse,—and a violent purging, which amounted to upwards of twenty evacuations in the course of twelve hours. The sutures were removed from the wound by Mr. Hutchison; the patient was turned on her side well over towards her front, so as to make the external wound depending, and, after which movement, two or three tea-spoonfuls of ill-conditioned pus immediately escaped from the wound.

Such decided relief from all unfavourable symptoms so quickly followed the change of position in this case, as to leave no doubt on the minds of Mr. H. and myself that the patient's life was in this instance saved chiefly by moving her round from lying on her back to her side, turned well over to her front, as before stated; and this is also the conviction of the patient herself. But it is at the same time correct to state, that, when the above dangerous symptoms first showed themselves, I was directed to bleed her to the amount of sixteen ounces; and some other remedies were at the same time prescribed, such as fomentations, &c.

The conclusion I have drawn from this case is, that, though adhesive inflammation soon closes the external wound, yet, from the unavoidable injury done to the parts (beneath the situation of the sutures) during the operation, in separating the hernia from its adhesions, suppuration will consequently take place, from the sides of the wound, not so closely kept in contact as the external labiæ or line of incision, and the matter must, by the patient lying on her back, fall into the cavity of the abdomen; and thence produce pain, peritoneal inflammation, and diarrhœa, as in the case I have just alluded to. And I am the more induced to believe that matter finding its way into the cavity of the abdomen in this case, was the sole cause of all the urgent symptoms that did occur, because they so immediately gave way to the treatment above adverted to,—namely, change of position.

We are favoured with but very few dissections after the operation for hernia when death has ensued; I should, therefore, consider it well worthy the attention of the practical surgeon to examine minutely, after the death of patients subsequently to this operation, into the causes of dissolution; and whether matter finding its way into the abdomen, be not frequently the cause of a fatal issue.

*21st April*, 1821.

